# The Vietnamese version of the Home Falls and Accidents Screening Tool (HOME FAST) - A preliminary study of validity and inter-rater reliability

**DOI:** 10.3389/fpubh.2023.1170000

**Published:** 2023-05-09

**Authors:** Lynette Mackenzie, Van Thanh Le, Doan Mai Ngoc Nguyen, Thy Hoang Phuong Dao

**Affiliations:** ^1^Discipline of Occupational Therapy, School of Health Sciences, Faculty of Medicine and Health, University of Sydney, Darlington, NSW, Australia; ^2^Rehabilitation Department, Faculty of Nursing and Medical Technology, University of Medicine and Pharmacy, Ho Chi Minh City, Vietnam

**Keywords:** accidental falls, home hazards, assessment, cultural translation, validity, expert panel

## Abstract

**Introduction:**

The Vietnamese Home Falls and Accidents Screening Tool (HOME FAST) was developed to measure the number of home hazards present in the homes of older Vietnamese people and the risk of falls.

**Methods:**

The HOME FAST and the HOME FAST manual were translated into Vietnamese by an independent translator and underwent backward translation by local health professionals into English to evaluate the accuracy of the translation. A panel of 14 Vietnamese health professionals evaluated the validity of the HOME FAST translation and rated the clarity and cultural relevance of each item. Ratings were evaluated using the content validity index (CVI). Reliability in ratings of the HOME FAST was evaluated using intra-class correlations (ICC), and ratings took place within the homes of two older Vietnamese people by six assessors.

**Results:**

In all, 22 out of 25 Vietnamese HOME FAST items were considered to have met content validity standards using the CVI. The ICC for home visit one was 0.94 (95% CI 0.87–0.97) and for home visit two was ICC 0.95 (95% CI 0.91–0.98) indicating high reliability.

**Discussion and conclusion:**

Bathroom items showed the most inconsistency in ratings indicating cultural differences in bathing activities. Descriptors of HOME FAST items will be reviewed for use in Vietnam to account for cultural and environmental differences. A larger pilot study is planned with older people living in the community in Vietnam to include calendar ascertainment of falls to determine if home hazards are associated with falling.

## Introduction

The population of Vietnam is aging with the proportion of older people predicted to grow from 11 to 28.5% by 2050 (1, 2). The prevalence of frailty is also increasing as the Vietnamese population ages, putting older people at risk of falls (3). Globally, falls in the older population is a major public health problem with numerous serious physical and psychosocial consequences that can lower quality of life (4). Vietnam is classed as a lower middle income country area (5), and a recent World Health Organization report (6) identified that globally, 75% of fatal falls among older people (aged 70 years and over) occur in low and middle-income countries. One Vietnamese study located in Danang found that 51% of their sample had reported a fall, and 64% reported fear of falls (7), therefore investigating falls risks is important to prevent falls in the home.

Added to this, Vietnam has an aging population with a declining fertility rate and increasing life expectancy (8). The fastest population growth is for older people over the last 30 years, and life expectancy from aged 60 of 20 years is similar to Thailand, Indonesia and Malaysia (9). Furthermore, the proportion of older people living with younger family members is decreasing, and while poverty has declined, a proportion of older people continue to live in poverty, especially in rural areas (9). Overall, the population in the ASEAN region is growing at an accelerated rate, with the population of 649 m outpaced only by China and India (10). The aging population of south-east Asia is predicted to surpass the proportion of the aging populations in North America and Europe (11).

Falls will become a major health issue in Vietnam as the population ages as age is a key risk factor for falling (12). Published studies on falls in older people is limited in Vietnam–for instance in a review of 15 randomized controlled trial falls studies in Asia there were none identified from Vietnam (13). In a scoping review of falls studies in south-east Asia (11), two published studies out of 37 were from Vietnam. One was retrospective observational study (14) and the other was a longitudinal intervention study (15). Both studies evaluated the relationship between visual problems and falls. The study reported female gender and living alone as significant factors. The interventional study found that corrective cataract surgery was effective in reducing subsequent falls.

The Home Falls and Accidents Screening Tool (HOME FAST) consists of 25 items related to features of the home environment and an older person’s functioning within the home (16). Each item is rated according to its capacity to put an individual at risk of falling. The HOME FAST was designed to be used by a health professional who scores each item as a hazard or not. The number of hazards putting a person at risk of falls are counted and a score of online or over means a higher risk of falls (17). The HOME FAST compares well with other measures of the home environment and demonstrates good psychometric properties and clinical utility (18). However, it is unknown how the HOME FAST can translate linguistically and culturally to a Vietnamese population. There may be some key differences in the home environments for older people in Vietnam, such as types of flooring surfaces and lighting, and poorly maintained public environments (13), which will need to be evaluated as part of the translation process. To design interventions to prevent falls, it is critical to be able to identify the kinds of home hazards that exist for Vietnamese older people using a valid tool.

Therefore, this study aimed to (i) translate the HOME FAST into Vietnamese, (ii) evaluate the validity of the Vietnamese HOME FAST in terms of clarity and relevance, and (iii) evaluate the reliability of the Vietnamese HOME FAST.

## Methods

The study used mixed methods:

(i)*Forward and backward translation*

The HOME FAST was first translated from English to Vietnamese by an independent translator, and the Vietnamese authors examined any inconsistencies in the translation. The Vietnamese version was then back translated to English and an expert panel of authors and Vietnamese health professionals checked the final version to be tested (19).

(ii)*Validity assessment of the Vietnamese HOME FAST*

**Figure 1:**
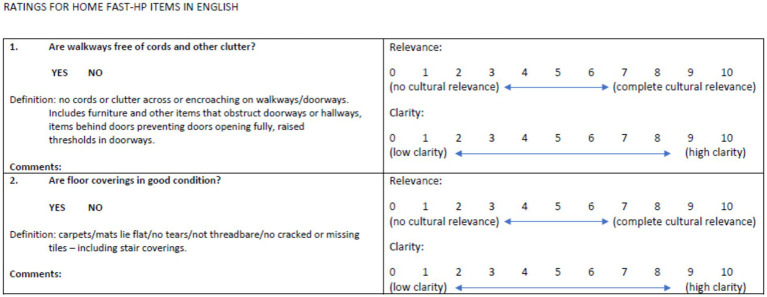
Sample of a survey item.

An expert panel of 14 Vietnamese speaking health professionals completed a survey to assess the linguistic validity of each of the items on the Vietnamese version of the HOME FAST (See Figure 1).

Ratings of 0–1.5 were considered not relevant/clear, 2–4.5 somewhat relevant/clear, 5–7.5 quite relevant/clear and 8–10 very relevant/clear.

The survey included ratings on the clarity and relevance of each of the items using a 0 to 10 Likert scale (e.g., 0 = low clarity, 10 = high clarity). Items in the Vietnamese version of HOME FAST were modified according to the written comments provided by the respondents. The content validity index (CVI) was used to evaluate the validity of each item. The CVI is the total number of experts giving a rating of 3 or 4 (relevant) divided by the total number of experts. When six or more experts are used, I-CVI values should not be less than 0.78 (20).

(iii)*Reliability assessment of the Vietnamese HOME FAST*

Two home visits were conducted with six health professionals independently completing the Vietnamese HOME FAST overall score for each one. The health professionals involved participated in a workshop to train them in the use of the HOME FAST prior to the home visits which was led by the developer of the tool who spoke English and an interpreter was used to ensure everyone understood the process of assessment. As there were more than two raters an intra class correlation (ICC_2_) was used (two-way random effects model) to evaluate agreement on ratings. The ICC was also appropriate as continuous scores were being compared.

Ethical approval was given by the University of Sydney Human Research Ethics Committee (Approval number 2020/018).

## Results

The final version of the Vietnamese HOME FAST was agreed and is available at https://stopfallsathome.com.au/resources/.

### Validity of the Vietnamese HOME FAST

**Figure 2:**
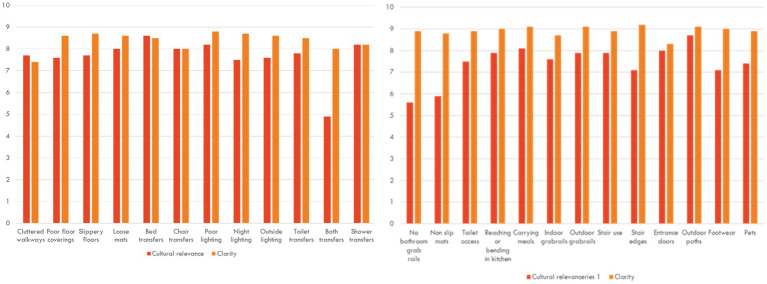
Scores for clarity and relevance of each HOME FAST item.

While all items were scored over seven for clarity, cultural relevance had lower scores for bathroom items (bath transfers, grab rails in the bathroom and use of non-slip mats) – see Figure 2. The content validity index (CVI) for each HOME FAST item is presented in Table 1. Only three items did not meet the usual threshold of a CVI of 0.78.

**Table 1:** Content validity index for HOME FAST items.

Cluttered walkways (1.0)	Outdoor step rails (1.0)	Shower transfers (1.0)
Loose mats (1.0)	Bed transfers (1.0)	Outdoor paths (1.0)
Toilet transfers (0.93)	Slippery floors (0.93)	Entrance doors (0.93)
Chair transfers (0.93)	Toilet access (0.93)	Reaching/bending (0.93)
No night lighting (0.93)	Carrying meals (0.93)	Poor floor coverings (0.86)
Stair edges (0.86)	Stair use (0.86)	Poor lighting (0.86)
Indoor step rails (0.86)	Pets (0.86)	Outside lighting (0.79)
Footwear (0.79)		
Non-slip mats (0.77)	Bathroom grab rails (0.77)	Bath transfers (0.57)

### Reliability assessment of the Vietnamese HOME FAST

For the first home visit conducted with six raters the total HOME FAST scores given by raters ranged from 8 to 14 out of 25 items. The percentage agreement for each HOME FAST item ranged from 67 to 100%, and there was 100% agreement for 15 HOME FAST items. For the second home visit, also conducted with six raters (not the same raters as in the first home visit) the total HOME FAST score ranged from 11 to 15 out of 25 items. The percentage agreement for each item ranged from 67–100%, and there was 100% agreement for 14 items. The bathroom items with the low CI scores identified in Table 1 also demonstrated inconsistency in rating from the home visits.

The ICC for the HOMEFAST overall score conducted at home visit one among six raters was 0.94 (95% CI 0.87–0.97) and for home visit two was ICC 0.95 (95% CI 0.91–0.98) indicating high inter-rater reliability.

## Discussion

This study aimed to develop and evaluate the validity and inter-rater reliability of the Vietnamese version of the HOME FAST. These preliminary findings confirm the utility of the translated version of the Vietnamese HOME FAST and its use in practice as a valid and reliable tool by health professionals. Further larger studies are needed to fully evaluate the psychometric properties of this tool with a Vietnamese population.

In the developed world, where most of the research on falls has taken place, there is evidence that unsafe home environments are associated with an increased risk of falls for older people, and that modifying the home to remove hazards can reduce falls (21–23), although there is less evidence in Asian settings and Vietnam in particular (11, 13). Falls can have serious consequences in terms of injury, cost and future care (22), and being able to measure hazards in the home associated with the risk of falls is essential to implement interventions to prevent falls. The HOME FAST is one such measurement and has been recommended by systematic reviews (18, 24). The HOME FAST has evidence of reliability (25), validity (26), responsiveness and predictability with respect to future falls of older people (27). The scoring of the HOME FAST has also been validated (17). This work was conducted in developed countries, therefore making the HOME FAST available in Vietnamese is the first step in developing evidence-based fall prevention strategies in Vietnam, rather than depending on more informal non-standardized ways of evaluating falls risk in the home for Vietnamese older people.

The findings of this study indicated there were cultural differences in the use of the home environment, such as the lower CVI for bathroom-related items such as the use of a bath, bath rails and bathmats, and a low score given for the relevance of bath transfers. Home environmental factors vary according to geography, culture, and architectural design (18). During these two initial home visits, it was obvious that bathtubs were not a usual feature of traditional Vietnamese homes and showers were much more frequently used. This would account for the low scores for cultural relevance and the low CVI for these items. As there is a not-applicable option on the HOME FAST for these items, this could be used in homes where there is no bathtub, and the structure of the HOME FAST can be preserved. More homes in the future might be expected to be built with Western-style bathrooms therefore, making these items more relevant. These findings are consistent with other studies where the HOME FAST has been translated and evaluated in other languages and cultural settings such as Chinese (28), Persian (29), Bahasa Melayu, Mandarin, and Tamil (30). No items were removed from the HOME FAST in these translated versions (30). Another study translated the self-report version of the HOME FAST into Mandarin for use in Hong Kong (31) and removed three items that were not considered culturally relevant to crowded living conditions in Hong Kong and merged two more items resulting in 20 items for the translated version. In this case a self-reported version may have required more clarification for older people to interpret the items themselves resulting in changes to the HOME FAST items.

The scores given for the two home visits indicated that with the exception of one rating the overall HOME FAST scores were nine or over, suggesting that the older people assessed were at high risk of falls (17). The inter-rater reliability findings from this study were very positive, and the ICC results were better than a previous Australian study evaluating the reliability of the HOME FAST (25) that reported an ICC of 0.82 (95% CI, 0.66–0.91) for the overall HOME FAST score. Training of raters is an important component of maximizing inter-rater reliability in the use of an assessment tool (32). A training manual for the HOME FAST is available at^1^ and was also translated into Vietnamese for this study. Face to face training sessions were also undertaken prior to the home visits, which may have contributed to the ICC findings. Previous studies have also suggested that effective home environmental assessments and modifications should be conducted by occupational therapists to prevent falls in older people (21–23). In Vietnam, the occupational therapy profession is in its initial development phase, so there is a shortage of appropriate health professionals who can undertake screening of older people at risk of falling. The HOME FAST was developed to allow any trained health professional to undertake an assessment of falls risk in the home of older people (33). A Vietnamese version of the HOME FAST has provided a tool that can be used in health professional practice in Vietnam and can be used as an outcome measure in ongoing research. The tool is tool now ready to be applied in a large study to further assess the validity, responsiveness and predictability of home hazard identification related to subsequent falls in Vietnam.

## Conclusion

The availability of a Vietnamese HOME FAST will now allow the home environments of older Vietnamese people to be evaluated according to their risk of falls. A larger pilot study is now planned with older people living in the community in Vietnam. The use of the Vietnamese HOME FAST will allow health professionals to identify home environmental risks and which features need to be modified.

## Data availability statement

The raw data supporting the conclusions of this article will be made available by the authors, without undue reservation.

## Ethics statement

The studies involving human participants were reviewed and approved by the University of Sydney Human Research Ethics Committee. Written informed consent for participation was not required for this study in accordance with the national legislation and the institutional requirements.

## Author contributions

LM and VL contributed to conception and design of the study, and organised the ethics approval. LM undertook the funding application, organized the database, performed the statistical analysis, and wrote the first draft of the manuscript. VL, DN, and TD undertook the forward and backward translation activities, organized the training for the raters, and the home visits. All authors contributed to manuscript revision, read, and approved the submitted version.

## Funding

The project received seed funding from the Vietnam Initiative of the Sydney South-East Asia Centre at the University of Sydney, Australia.

## Conflict of interest

The authors declare that the research was conducted in the absence of any commercial or financial relationships that could be construed as a potential conflict of interest.

## Publisher’s note

All claims expressed in this article are solely those of the authors and do not necessarily represent those of their affiliated organizations, or those of the publisher, the editors and the reviewers. Any product that may be evaluated in this article, or claim that may be made by its manufacturer, is not guaranteed or endorsed by the publisher.

## Footnotes

^1^https://hdl.handle.net/2123/29645
